# A community context for aggression? Multi‐species audience effects on territorial aggression in two species of Paridae

**DOI:** 10.1002/ece3.7421

**Published:** 2021-04-03

**Authors:** Jin Bai, Todd M. Freeberg, Jeffrey R. Lucas, Kathryn E. Sieving

**Affiliations:** ^1^ Department of Wildlife Ecology and Conservation University of Florida Gainesville FL USA; ^2^ Department of Psychology University of Tennessee – Knoxville Knoxville TN USA; ^3^ Department of Biological Sciences Purdue University West Lafayette IN USA

**Keywords:** heterospecific audiences, Paridae, playback experiments, songbird behavior, territorial aggression

## Abstract

1. Territorial aggression in birds is widely observed and is commonly linked to sex, age, body size, physiology, seasonal cues, food resource, urbanization, and a variety of social contexts including conspecific audience effects. However, little is known about the heterospecific audience effects on territorial aggression.
2. Here, we address an emerging idea that heterospecific audience effects may be pervasive influences in the social lives of free‐living birds. We tested the hypothesis that the composition, number, and relative body size of heterospecific audiences observing an aggressive contest will influence the response probability and intensity of aggression displayed.
3. We subjected two Paridae species, tufted titmouse (TUTI, *Baeolophus bicolor*) and Carolina chickadee (CACH, *Poecile carolinensis*), to playbacks of aggressive calls during a breeding season in north‐central Florida. At widely spaced playback sites (*N* = 134) in woodland habitats, we characterized the makeup of heterospecific audiences, aggression type (intra vs. interspecific territoriality), local population density, and various environmental factors (tree density, wind speed, and noise level) that are likely to influence territorial aggression.
4. We found that the presence of heterospecific audiences increased TUTI aggression levels and that both parids were more likely to respond to playback stimuli when their audiences had higher heterospecific diversity (more heterospecific individuals and species). We also found TUTI were more likely to respond when CACH were present but not vice versa.
5. In conclusion, we found evidence that heterospecific audiences significantly influenced the metrics of territorial aggression of free‐living animals and we suggest that the definition of audience effects on the behavior of free‐living animals be expanded to incorporate heterospecific audiences.

## Graphical abstract

We reported territorial aggression behavior of two Paridae species, tufted titmouse (TUTI, *Baeolophus bicolor*) and Carolina chickadee (CACH, *Poecile carolinensis*). We found that the presence of heterospecific audiences increased TUTI aggression levels and that both CACH and TUTI were more likely to respond to playback stimuli when their audiences had higher heterospecific diversity.

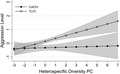

## 1 INTRODUCTION

Aggression is ubiquitous across animal taxa, and a variety of factors affect the outcome of contests including both endogenous (e.g., hormone titer, risk perception, experience, age, sex, body size, and personality; Basset & Angelis, 2007; Fedy & Stutchbury, 2005; Fuxjager et al., 2017; Whittaker et al., 2018) and exogenous stimuli (e.g., resource access, weather, noise, seasonal context; Demeyrier et al., 2017; Fuxjager et al., 2017; Mac Nally & Timewell, 2005). The immediate social context of aggression matters to aggressors and can be explained in terms of audience effects. Audience effects on aggressive behavior occur when nearby eavesdroppers of conflicts influence the aggressors' behavior. For example when a female observes male–male conflicts, the males' aggression can escalate (Bertucci et al., 2014; Ronald et al., 2015; Szipl et al., 2018). A related term, bystander effect, defines two different situations where the eavesdroppers at a conflict are themselves influenced either by (a) each other's behavior (the presence of other bystanders can repress helping or intervening in the fight; Darley & Latane, 1968; Havlik et al., 2020), or (b) by the outcome or dynamics of the fight itself (e.g., losers are often comforted by bystanders; Fraser et al., 2009). It is possible that conspecific bystanders may improve their future social standing by attending conflicts and contestants may adjust their aggressive behaviors depending on audience makeup to achieve similar functional benefits in social standing (Baltz & Clark, 1997; Fedurek et al., 2015). Bystander effects of either type are not the focus of this study, and we want to evaluate the audience effects on aggressors' behavior other than the influences bystanders received.

Audience effects are traditionally limited to conspecifics, but increasingly we understand that the relevant social context for numerous animal behaviors includes heterospecifics, and this is exemplified in avian ecology. When mobbing a predator (Krams & Krama, 2002; Nolen & Lucas, 2009; Sieving et al., 2004), foraging (Dolby & Grubb, 1998; Goodale et al., 2020; Suzuki & Kutsukake, 2017), finding breeding habitat (Jaakkonen et al., 2015; Szymkowiak et al., 2017), or assessing predation risk (Hetrick & Sieving, 2012; Seppänen et al., 2007), birds are heavily influenced by other species' presence, activities, and social information conveyed in vocalizations. Indeed, heterospecifics in the same trophic level can be as or more important in facilitating survival under predation pressure than conspecifics (Dolby & Grubb, 1998; Goodale & Kotagama, 2008; Jones & Sieving, 2019; Slagsvold & Wiebe, 2017). Species may eavesdrop on key information from sympatric heterospecifics (Grade & Sieving, 2016) or find protection from attack by associating closely with more numerous, stronger, more vigilant, or more aggressive species (Goodale et al., 2020). All such behaviors involve a broadly heterospecific social context within finely tuned communication networks of species that produce or eavesdrop on sets of signals perceived as biologically relevant by all (Jones & Sieving, 2019; Sieving et al., 2010).

Socially aggressive interactions in animals typically involve conspicuous signaling between rivals—from loud vocalizations, stereotypical visual displays, and deadly attacks to subtle somatic color changes (Carlos, 2006; Deckel & Jevitts, 1997; Kalinoski, 1975; Searcy et al., 2006; Sieving et al., 2000). Like mobbing displays, the vigor of territorial displays may be calibrated to the perceived risk of attack by nearby predators (Akçay et al., 2016; Hua et al., 2016). Therefore, if mobs attract heterospecifics as sources of useful information about predation, threat locations, threat abatement, or signaler quality (Dutour et al., 2016; Gintis et al., 2001; Goodale et al., 2019; Hurd, 1996), conspicuous social contests may also provide similar, overlapping, or otherwise unique informational and risk management resources to participants (Seppänen et al., 2007; Sullivan, 1984). Alternatively, if multiple neighborhood species share the same geographical boundaries with their own conspecific competitors, then a territorial dispute by one species may offer an opportunity for synchronous reinforcement of everyone's conspecific boundaries, with minimal collective risk of predator attack (Goodale et al., 2019; Munn & Terborgh, 1979). Regardless of the motivation to gather, eavesdroppers attracted to a fight should influence the perceived risk of attack during a conspicuous encounter, thereby modifying the intensity of agonistic display (audience effects; da Cunha et al., 2017).

We have noted over the years that heterospecific audiences are often attracted to and observe conflicts between passerine territory holders (see also De La Hera et al., 2017). Aggression research is abundant and well‐integrated across many endogenous and exogenous phenomena, but heterospecific social contexts are typically ignored (Trainor & Marler, 2010). One of the persistent questions in aggression research is identifying contextual conditions that can reduce or increase its intensity (Fuxjager et al., 2017; Huntingford, 1976). We suspect that within multi‐species interaction networks, heterospecific audience effects may be a significant modifier of aggression intensity. Therefore, we hypothesized that the makeup (composition, number, relative body size) of heterospecific audiences observing a fight should significantly influence the level of aggression displayed.

To test this hypothesis, we chose two sympatric species in Family Paridae (referred as parid throughout the text): tufted titmice (TUTI: *Baeolophus bicolor*) and Carolina chickadees (CACH: *Poecile carolinensis*) that are native year‐round residents in wooded habitats of North‐central Florida and much of the Eastern United States (Harrap & Quinn, 1995; Otter, 2007). We selected these species for two main reasons. First, both species participate in mixed‐species groups in winter, sometimes together, and serve as keystone information providers to a wide array of species that share predators with them (Contreras & Sieving, 2011; Farley et al., 2008; Jones & Sieving, 2019). Also, both are dominant alarm callers at predator mobs (Nolen & Lucas, 2009; Sieving et al., 2004). Therefore, we expected the territorial disputes of these two species should (and do) attract heterospecifics, providing an opportunity to explore heterospecific audience effects on aggression intensity. Second, we were interested in clarifying the level of interspecific aggression between these two parids that are often closely associated in winter flocks (Harrap & Quinn, 1995; Morse, 1970). Titmice dominate chickadees at feeders and in foraging flocks in winter, yet they also interact in obtaining novel foods (Cimprich & Grubb, 1994; Freeberg et al., 2017) and share the same antipredator signaling system (Hetrick & Sieving, 2012). Moreover, both species can kill the other in direct conflicts despite a large size difference (titmice ~ 20–25 g; chickadees ~ 10–12 g) yet can also peaceably coexist in aviaries together (Coppinger et al., 2020).

We generated aggressive responses to playback stimuli within and between the two species and observed birds in attendance during respondents' aggressive displays. We expected that more diverse or perhaps larger‐bodied audiences would increase aggression intensity between conspecifics by reducing the perceived risk of predatory attack during conflicts. We also expected that the larger titmouse would show greater aggression toward chickadees than vice versa and that both titmice and chickadees would be most vigorous in conspecific contests. We tested for the different influences of (a) inter‐ or intraspecific aggression, (b) presence of the nontarget parid species, (c) presence of larger nonparid heterospecifics, and the (d) diversity of species and number of individuals composing the audience on respondents' aggression intensity. In sampling design and analysis, we controlled for a variety of potentially confounding factors known to influence aggression including tree density, noise level, and local territory densities of the study species occupying the study sites.

## 2 METHODS

### 2.1 Study sites

Fieldwork took place at Ordway‐Swisher Biological Station (OS: 29.69°N, 81.98°W), San Felasco Hammock Preserve State Park (SF: 29.74°N, 82.45°W), and suburban/urban habitats within the City of Gainesville (UB: 29.64°N, 82.35°W), Florida, USA. Pine flatwood communities dominate the habitats of OS where tree density is naturally lower than the mesic and xeric hardwood habitats of both SF and UB (Stout & Marion, 1993; Ware et al., 1993). The playback experiments were conducted between 08:00 and 12:00 (Eastern Standard Time) during an avian breeding season in North‐central Florida from February to August 2018 (most data were obtained from May to July). In total, 134 playback trials were conducted (OS: *n* = 45; SF: *n* = 44; UB: *n* = 45; see Supplementary Online Materials, Figure S1 for playback locations).

### 2.2 Stimulus design

In designing the auditory stimuli, we acknowledged that natural aggressive encounters in birds are often escalatory (Fuxiager et al., 2017; Hof & Podos, 2013). Therefore, to mimic natural intrusions in order to keep respondents engaged up to their highest level of aggression, we designed our stimulus to present calls representing a scale of aggression intensity akin to interactive playbacks (King, 2015), a technique that can elicit extreme aggression in birds. Levels of aggression in both study species are readily identifiable by the production and acoustic structure of different call types they use (Hailman, 1989; Hetrick & Sieving, 2012; Offutt, 1965) and by nonvocal behavior (Foltz et al., 2015; Hardman & Dalesman, 2018; Nolen & Lucas, 2009).

Each playback recording included four sections (2 min/section), each with a mix of calls typical of low, medium, high, and extreme aggression intensity (described below, Table S1). We used three equivalent replicate playback sound files (randomly selected for use in each trial) to broadcast the calls of each of the two species (six total playback recordings). Replicate sound files were structured similarly but used different vocal resources to avoid stimulus pseudoreplication. We extracted vocal material from high‐quality field and aviary recordings collected from populations in North‐central Florida (Hetrick & Sieving, 2012; Sieving et al., 2010) and from Xeno‐canto resources recorded in Florida (Xeno‐canto, 2018). We used methods from previous studies to implement interactive playback methods with both species to arrange the escalatory sequences of call types for playback (Hailman, 1989; Lamica & Sieving, 2017; Smith, 1996). We constructed playbacks to mimic an escalation of aggression from simple territorial advertisement and contact calls up to vocalizations associated with imminent physical aggression (e.g., a passively constructed escalatory playback sequence; Dabelsteen & McGregor, 1996). The background noise in each playback was high pass filtered at 1 kHz. Each WAV file track was constructed using Adobe Audition CC 2014. The number of calls that were used in the playback is shown in Table S2.

Each species has a distinct territorial advertisement song (“feebee” for CACH; “peter‐peter” for TUTI; Pieplow, 2017) given both within and outside of direct aggressive conflict. In addition, both parids share two classes of calls: soft seets (contact calls) and “chick‐a‐dee” calls. The former are single notes that are nearly indistinguishable between the two species and usually signify unstimulated states of foraging and family cohesion (Sieving et al., 2010). The latter call type is very well characterized for both species because it is a complex call that can encode a rich array of graded signal content related to pair and group cohesion, aggression, antipredator signaling, and food‐related information (Krams et al., 2012; Lucas & Freeberg, 2007; Sieving et al., 2010). Chick‐a‐dee calls vary in the number and kind of notes in the initial “chick” and subsequent “dee” section in species‐ and context‐specific ways as aggression toward predators or competitors escalate. For contexts of aggression at the higher end of escalation, both species have a unique set of species‐specific call types that come into play (squeal and flutter display call for TUTI; gargle call and variable see‐strings call for CACH; Figure S2; Baker & Gammon, 2007; Mostrum et al., 2002; Ritchison et al., 2015).

Given this set of call types, we populated the first 2 min section of recordings with “soft seets” and short duration (low or no‐aggression) chick‐a‐dee calls; the latter with <5 dee notes in the dee section of the call. Both call types are characteristic of quietly foraging family groups of both species that we hoped would stimulate curious attention by target respondents. The next three sections increased aggression intensity by adding songs (sections 2, 3, and 4) and other aggressive calls (squeal and flutter display calls in TUTI tapes, and gargle calls in CACH tapes; sections 3 and 4). We designed these 8 min tapes (digital sound files) in this way to draw out a graded response allowing initiation of respondent aggression toward a stimulus exhibiting, at first, only simple intrusion by foraging birds, up to highly escalated aggression in response to calls known to be used when contests may become physical. Switching calls during playback invites call matching (Burt et al., 2001) and prevents habituation as well as stimulating escalation of aggression (Hof & Podos, 2013).

### 2.3 Playback trials

We randomly selected playback locations ahead of time on previously mapped transects distributed across the study sites. To insure independence of samples, random points sampled within the same day were at least 200 m apart, and those sampled on different days were much further apart. Moreover, when walking from one trial to the next on the same day, observers tracked previous respondents' locations (by their calls) until the calls could not be heard anymore before setting up the next trial to insure that only new birds were ever used. All trial locations were used one time. Once arriving near the selected location, we identified the respondent by observing an individual or hearing its call within 30–50 m. We set up a speaker (Kunodi F4) on a tree branch at 2 m height but occasionally on the grass in urban areas if constrained by private property lines. The speaker was connected through Bluetooth from an iPhone X with a standardized volume (the first author maximized the volume on the phone and kept the speaker at 3/4 maximum volume). We tested the max and min SPL (dBA) of our playback at 5 m from the speaker (open forest: CACH 31.1–75.9 dB, TUTI 31.5–70.9 dB; dense forest: CACH 32.2–77.0 dB, TUTI 32.5–67.9 dB; urban street: CACH 31.1–84.6 dB, TUTI 35.8–72.9 dB), and we confirmed that we could hear even the softest stimuli from the 20 m max distance. In all cases, ample perching substrate was available directly above the speaker. We placed a Marantz PMD 661 MKII digital recorder with a Sennheiser ME67 Shotgun microphone on a tripod at least 2 m from the speaker. Once a randomly chosen exemplar of the treatment was selected (intraspecific or interspecific), the observer (the first author in all cases) waited for 60 s before starting the playback while quickly using a range finder to locate markers (four trees in four directions) around the edge of a 20 m radius around the speaker. This 1 min wait did not include any behavioral measurements, and the only purpose of this period was to mark the edge of the observation circle and standardize the range of the observation circle (to determine when and if a bird was in the observation circle during a trial). After the 1 min wait, if the target bird was still present at the observation circle (either identified by sound or sight), the playback trial proceeded. No trials proceeded without confirmation of the presence of the target bird.

At each trial location, two treatments could occur at the same time due to the presence of both species (singular playbacks: TUTI alone, *n* = 67; CACH alone, *n* = 18; simultaneous playbacks: *n* = 49). For example, when chickadees received the treatment of titmice playback, the titmice close to our target chickadees could also respond to playback. We tracked both treatments at the same time as two playback trials representing a chickadee interspecific trial and a titmouse intraspecific trial. All singular and simultaneous playback trials were included in the data analysis. The variable “other parid presence” accounts for the potential influence of the presence of titmouse on the targeted chickadee's interspecific aggression as well as the influence of the presence of chickadee on the titmouse's intraspecific aggression.

When the stimulus playback finished at 8 min, the observer (the first author in all cases) continued recording aggression measurements for up to 10 more minutes for a maximum observation time of 18 min (only a few highly aggressive individuals reached the maximum). Thus, we defined a response period of up to 18 min, comprising two parts, including individuals' response when the playback was on (8 min) and individuals' response when the playback was off (max 10 min). This period of 18 min did not include a brief setup period for marking the edges of the observation circle.

### 2.4 Response outcomes

We were interested in two different results. First, we assessed factors affecting response status or whether a nearby target that we detected prior to setup actually came within 20 m of the stimulus to exhibit aggression. Second, if the target bird did approach, we assessed factors determining the aggression intensity level the responding species exhibited. We used two different analyses for each outcome.

### 2.5 Aggression metrics

We conducted seven measurements of aggression intensity (six taken during playback trials): (1) latency to start responding, (2) the number of flights per bird, (3) the number of calls per bird, (4) the closest approach distance, (5) total time spent, (6) latency to stop responding, and (7) response group size. During playback trials, latency to start (seconds) was recorded as the time between the start of the playback and the first vocalizations or movements toward the speaker. The number of flights per bird (counts) included flights between trees, flights between branches within a tree, flights toward the speaker, and the audible movement of opening wings in the air (if the movement was masked by leaves and very close to the observer). The number of calls per bird and call types were enumerated postplayback from recordings of every playback sequence using Adobe Audition CC 2014. To separate respondent's calls from stimulus calls, both the stimulus recording and the recording taken during playback were directly aligned in two spectrographic panels such that respondent vocalizations could be distinguished from broadcast vocalizations. Measurements of the number of calls and flights were summarized for all respondents of the target species occurring at a sample point, then divided by the number of responding individuals of the target species. For the closest approach (meters), we recorded the minimum horizontal distance between the speaker and the bird using a laser range finder when the bird's closest approach was located in another tree (>5 m). When a bird's closest approach was at the same tree of the speaker or the tree next to it (horizontal distance is <5 m), we used measuring tape to measure the exact distance. Approach distances were measured after the playback trial by memorizing the closest approach location (e.g., branch or ground surface). Latency to stop responding (seconds) was defined as the time between the end of playback and the time when birds disappeared outside the 20 m radius or birds became silent for at least 1 min without approaching the speaker. Total time spent responding (seconds) was the difference between when the target species first called or moved toward the speaker and the end of the aggressive territorial response. Response group size was the number of conspecific individuals that responded to the playback at each trial. We used response group size as one of the aggression metrics because we were measuring aggression level at the playback trial level other than individual level. We did not mark or band any individual, so we could not track one individual's aggression while treat the number of other conspecifics as a predictor for aggression. The aggression level at each playback trial came from all individuals that were participated. These measurements described above were used to create a single measure of aggression intensity (details were described in the section of data analysis). We also want to clarify the logistics of the data collection for aggression measurements. The first author conducted all playback trials and collected all the data in this project. The number of flights per bird and response group size was the only variables that required real‐time tracking during each playback trial. Latency to start, latency to stop, and total time spent were measured at the beginning and the end of playback trial. The closest approach distance was measured immediately after each playback trial. The number of calls per bird as described above was measured in the laboratory using computer software.

### 2.6 Environmental metrics

Immediately after trials, we measured average and maximum noise levels (dB) using a phone application Decibel X (version 7.0.0). We ran the app for 30 s with an iPhone X placed on a tripod (1.5 m from the ground) and recorded the mean and maximum noise levels for relative comparisons of background noise in analyses. Acknowledging that a commercial meter may more accurately measure actual sound pressure levels (SPL), the iOS platform we used is superior to other smart phone applications, capable of providing estimates within 1 dBA of true SPL (Murphy & King, 2016). Since we were not interested in accurate measures of SPL but rather reliable relative measures for comparison across our study sites, this methodology was satisfactory for our needs. For the measurement of basal area, we used an angle gauge (Brady, 1995) to estimate the area (m^2^) of tree stems at breast height surrounding the observer. We also quantified the percentage of forest canopy cover surrounding each playback point as another measure of tree density. To do this, we located each sample point on Google Maps, scaled each image to 350 m aerial height, and took a screenshot. By overlaying a transparent quadrat with 10 by 10 grid squares (Figure S3, 100 squares total), we counted the number of squares occupied by more than 50% canopy cover, calculating percent canopy cover as (# of significantly occupied squares/100 squares) * 100. Aerial images were taken in 2018 within one month of sampling, and no major logging, fires, or other canopy disturbing events occurred in this time at any of the sites. Wind speed was measured using a “Kestrel 3000 Weather Meter” immediately after each playback trial.

### 2.7 Audience metrics

To account for the immediate social context of parid‐on‐parid aggression, we assumed that the number, diversity, and/or sizes of audiences could influence the level of aggression displayed during contests. To characterize the audience in attendance at a playback, we counted the total number of heterospecific individuals present within 20 m of the speaker at any point (for any part or all) of the playback period. In addition to the number of species and individuals of parids and nonparids in attendance, we used “Birds of North America Eastern Region” to extract each species' mean weight (Table S3; Vuilleumier, 2011). To characterize the relative sizes of heterospecifics in relation to each of the two parid species, we classified each heterospecific as either similar to, smaller, or larger than the target parid. For chickadees (mean mass = 11.0 g), similar or smaller sized heterospecifics weighed < 16.0 g and larger heterospecifics weighed > 16.0 g. For titmouse (mean mass = 20.0 g), similar or smaller sized heterospecifics weighed < 25.0 g and larger heterospecifics weighed > 25.0 g. If there were one or more than one larger bird present, then the trial was categorized as having larger heterospecifics (1). If all birds were similar or smaller sized than the target species, the trial would be categorized as no larger heterospecific in attendance (0). This approach was justified by the following. Raptors choose the largest prey available that they can handle (Gotmark & Post, 1996). Assuming all species in our sample are prey for the Cooper's hawk (*Accipiter cooperii*)—the most common, to these small songbirds, and lethal diurnal bird predator in the area—then noticeably larger birds in attendance at a fight should have been more attractive to a hunting hawk than the small parids (Sieving et al., 2010). Therefore, we predicted that more and larger heterospecifics in the audience would “release” greater, more conspicuous aggressive behavior in respondents. A key assumption is that the parids can perceive the greater safety afforded by the presence of larger/more species (dilution effect; Beauchamp, 2017).

### 2.8 Population metrics

To determine whether local parid densities would influence aggression levels, we used transect‐based counts of parids (three 1 km transects each in OS and SF censused during January 2017, and six total 1 km transects in UB; three censused in October–November 2017 and three during January 2018; Cubaynes et al., 2014). Proximity at the closest points on any two transects was at least 500 m. We broadcast territorial playback of each species for a minimum of 10 min total at every 100‐m marker (including the 0 and 1,000 m points). At each point, we recorded how many individuals of each species responded (coming within 50 m of the playback point) and excluded individuals that approached from the direction of the previous point (if we had encountered birds at the previous point) in the counts of total individuals encountered per transect (Bibby et al., 2000).

Diameters of territories were defined by the number of consecutive points defended by the same individuals identified as following us from one point to the next. In this manner, we could then calculate four different metrics reflecting territory density and the number of individuals per km of the linear transect. Measures included territory density (mean number of defended territories/km; Figure S4), linear population density (mean number of birds encountered/km), territory gap length (mean distance across consecutive undefended points), and territory width (mean distance that individuals followed observers to defend successive points; transect data summarized in Table S4). Stimulus source included all the recordings for each species provided in the iBird Pro Guide to Birds app (Version 10.06) and was broadcast using an iPhone 6s and battery‐boosted iHome Bluetooth speaker set to an average SPL level at 56.4 dB (range from 36.2 to 71.1 dB) at 1 m from the speaker.

Measurement of territory density occurred as a separate process from the aggression playback trials but was conducted in the same three large study areas. Transects were spaced and placed independently for each study area; a very few transects from density surveys were likely near aggression transects (study areas were large) but were used in different seasons with different stimuli. The purpose of the density measurement was to have a relative indicator of the “background” population density for each study area/habitat (urban forests, hardwood forests, and pine forests). Given our standardized survey protocol (same total distance surveyed, randomized placement of transects, same number of playbacks using the same taped call sets, etc.), the measurements of territories and birds per Km provide a comparable indicator across habitats. We measured territory density at all three study areas in one winter season but only two of the areas in the same breeding season, so we only used data from winter in this paper. Parids are year‐round residents who vigorously defend territory all year, as do many passerines even if they migrate to separate wintering areas (e.g., Brotons, 2000; Cuadrado, 1995). Even if density and responses are somewhat seasonal, the resultant density measures are used here merely as relative indicators of density.

### 2.9 Data analysis

#### 2.9.1 Aggression score

To define the aggression level of each responding individual, we used factor analysis (FA; Stata version 15) to generate independent and normally distributed aggression scores for analysis. After dropping latency to stop responding (due to lack of normalizing transformation), we used the remaining six for use in the FA (Table S5, Table S6, & Table S7). Only the first factor component had an eigenvalue > 1.0 (2.28); it explained 80.8% of the variation in the data, and in the manner we expected. A low aggression score indicated fewer target species individuals responded, they had a long latency to respond, they stayed relatively far away from the speaker, and vocalized and flew near the speaker only rarely. A high aggression score represented a larger responding group (mean group size = 2.35, *SD* = 1.23) that moved in quickly (mean latency to start = 168.78; *SD* = 149.94), came close to the speaker (mean closest approach = 6.2; *SD* = 5.15), vocalized more (mean number of calls per bird = 47.18; *SD* = 57.19), and flew frequently (mean number of flights per bird = 8.54; *SD* = 8.66) from perch to perch during the response periods.

#### 2.9.2 Data reduction

To represent covariables—factors known to influence aggression that were not central to our hypothesis—in our modeling, we used correlation analysis (pairwise Pearson; if coefficients were > |0.30| and *p* < 0.05, we deemed them to be significant) to identify clusters of colinear variables with functional relationships, followed by principal components analysis (PCA) on three distinct functional groups of variables to obtain uncorrelated components representing each group.

#### 2.9.3 Heterospecific diversity

Using principal components analysis (PCA) on the number of heterospecific individuals (min = 0, max = 17, mean = 4.7, *SD* = 2.97) and species (min = 0, max = 14, mean = 3.59, *SD* = 2.40) that came within 20 m of the playback center during the playback period, we obtained a single significant component representing heterospecific diversity of the audience (not including the other parid species; eigenvalue = 1.9, 95% variance explained). Loadings on the PC = +0.71 (individuals) and +0.71 (species).

#### 2.9.4 “Background” population density

Our playback methods for line transects of parid population density yielded several related measures characterizing background population density for each responding parid species, including mean territory density (# territories crossed/km; min = 1, max = 6, mean = 4.39; *SD* = 1.56), mean territory gap distance (# of meters between last and next contact with a responding bird/km; min = 127, max = 900, mean = 242.62; *SD* = 213.73), mean territory width (# meters between first and last contact with a bird following the observer from playback to playback/km; mean = 161.16; *SD* = 24.23), and mean population density (# of unique individuals detected/km; min = 131, max = 200, mean = 10.64; *SD* = 4.05). Ordination of these 4 measures across study sites in a PCA yielded a single significant component (eigenvalue = 3.05, 76% variance explained). Loadings on the PC = +0.53 (mean territory density), −0.52 (mean gap distance), −0.40 (mean territory width), and +0.54 (mean population density).

#### 2.9.5 Vegetation, wind, and background noise

Finally, avian aggression and vocal production can be influenced by each of these measures—responses decline with wind speed (Weeden & Falls, 1959), increase with noise level (Phillips & Derryberry, 2018), and increase or decrease depending on habitat quality, which for these birds, is tied to the density of hardwood trees (Harris & Reed, 2001). Moreover, these metrics are highly functionally related to each other—wind and anthropogenic noise are degraded with increasing forest vegetation density. The summary statistics for four measures we used (two for vegetation measures) were as follows: canopy cover (min = 22%, max = 100%, mean = 71%, *SD* = 0.24); basal area (min = 15, max = 200, mean = 83, *SD* = 39.25); wind speed (min = 0.0, max = 6.0, mean = 1.1, *SD* = 1.27); average noise level (min = 41.5, max = 77.5, mean = 55.1, *SD* = 7.08; all in dBA). A PCA detected a single significant component (eigenvalue = 1.87, accounting for 47% of variation). Loadings on PC = +0.58 (canopy cover), +0.54 (basal area), −0.41 (average noise level), and −0.46 (average wind speed). None of the three principal components derived above (parid population density, heterospecific diversity, or vegetation‐wind‐noise) were significantly correlated with each other in pairwise comparisons (*r* ^2^ < |0.30|; range from −0.0051 to −0.21).

#### 2.9.6 Climate effects

We did not include Julian date, temperature, or humidity in the models because they were not correlated with aggression in exploratory analyses, and we wanted to minimize noninfluential model terms if they were not of interest. Julian date was not of interest because parids are territorially aggressive all year which means that cortisol reactivity, rather than seasonal changes in testosterone largely control territorial behavior (Landys et al., 2010). Also, by sampling the three major habitats and all four treatment types with equal effort in each month through the entire sampling period, we minimized any confounding effects of time of year on the other factors. Mean morning temperature during sampling was consistently moderate (mean = 79.5°F, *SD* = 5.59), eliminating energetic constraints on exertion which are of concern in aggression studies at higher latitudes or across altitudinal gradients (Freeman & Montgomery, 2016), but not in our study.

#### 2.9.7 Hypothesis testing

For both aggression intensity (aggression factor score) and response status (playback generated a response or not) models, we used generalized linear modelling (maximum likelihood; Gaussian distribution with identity link, or Binomial distribution with logit link, respectively) with a standardized model comparison and reduction procedure. The full model for both analyses tested 28 terms total, including seven main effects: (1) responding species (CACH or TUTI), (2) aggression type (interspecific or intraspecific), (3) other parid presence (0 or 1), (4) presence of at least one larger heterospecific individual (0 or 1), and three principal components representing (5) heterospecific diversity in the audience, (6) local parid population density, and the (7) habitat descriptors (vegetation, wind speed, noise). Also included were selected 2‐way interactions (15 total) including all those possible 2‐way interactions except for those between the latter three main effects (principal components; 5–7). Six 3‐way interactions of interest included all possible combinations of main effects 1–4 (three terms) and three terms that included main effects 1 and 2 with each of terms 5, 6, and 7. We prioritized including both responding species and aggression type (main effects 1, 2) in interactions with the other variables because together they define the four playback treatments. To arrive at the best fit reduced model, the least significant interaction terms (based on the highest *p*‐values) were removed sequentially until all remaining interaction terms had *p* < 0.10. Interaction terms with *p*‐values > 0.05 were retained if marginal contrasts were significant. Akaike's information criterion (AIC) was used to ensure significant model improvement was achieved via model reduction (at least two points).

As in many ecological studies that occur in the field, we had defined hypotheses represented by our main effects terms, and a variety of potentially influential factors gleaned from the literature that we measured. In order to prioritize the hypothesis testing, all main effects were retained in final models to account for their effects as we planned. The AIC metric we used accounts for parsimony, or reduction in the number of terms, represented by AIC = −2 * LL + 2 * *k* = −2(LL − *k*) where LL = model log‐likelihood and *k* is the number of predictors (2 * *k* is a penalty term). In this way, our model selection allowed us to jettison unimportant covariables and interactions from final models, reducing 28 terms to 8–12 final terms in our best fit models of interest. Because we were hypothesis testing and not “fishing” for influential terms, we took the best fit model as the most parsimonious test of those hypotheses rather than presenting a candidate set of best ranked models, or by model averaging (Symonds & Moussalli, 2011). All analyses were conducted in STATA version 16.

## 3 RESULTS

### 3.1 Response status

**FIGURE 1:**
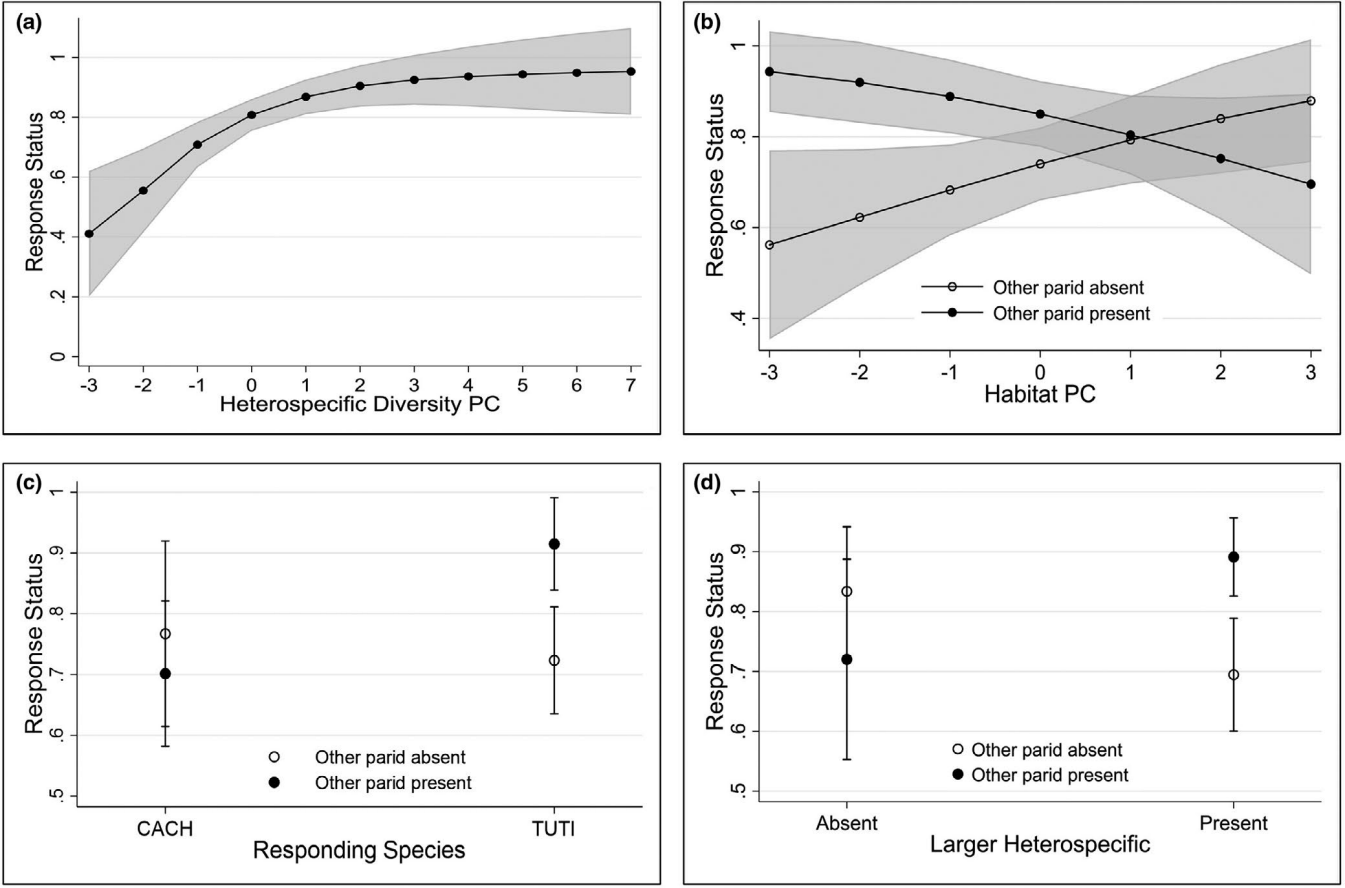
Predicted probability of response status at playback trials; the target species approached (1) or did not approach (0) as defined by (a) the diversity of heterospecifics audiences observing the trial; (b) a two‐way interaction between the presence or absence of the other (nontarget) parid species and the habitat condition at the playback trial.; (c) a two‐way interaction between responding species and the presence or absence of the other (nontarget) parid species; (d) a two‐way interaction between larger heterospecific presence and the presence or absence of the other (nontarget) parid species. Heterospecific diversity in (a) represents the number of heterospecific individuals and the number of heterospecific species during the playback trial. The *x*‐axis of Habitat PC in (b) represents basal area, canopy cover, noise level, and wind speed (increasing score indicates higher tree density and lower noise level and wind speed). Responding species in (c) include CACH (Carolina chickadee) and TUTI (tufted titmouse). All confidence intervals set to 95%

Based on our best fit model on response status and the contrasts of predictive margins on significant interaction variables (Tables 1 & Table S8‐S9), the likelihood of responding to playback significantly increased when the heterospecific diversity score is higher (Coef = 1.866, *p* = 0.001; Table 1 & Figure 1a). Furthermore, interspecific playback was significantly less likely to have a response than intraspecific playback (Coef = −3.159, *p* < 0.001; Table 1 & Figure S5). The interaction between other parid presence and responding species showed a significant effect on response status (Coef = 2.431, *p* = 0.030; Table 1). Other parid presence significantly increased the likelihood of aggression responses from TUTI (Coef = 0.192, *p* = 0.001; Table S8 & Figure 1c). The interaction between other parid presence and larger heterospecific presence also showed a significant effect on aggression response (Coef = 2.825, *p* = 0.019; Table 1). When a larger heterospecific was present, other parid presence significantly increased the likelihood of aggression response (Coef = 0.197, *p* = 0.001; Table S8 & Figure 1d). Furthermore, the interaction between other parid presence and habitat conditions (vegetation/wind/noise levels) showed a significant effect on aggression response (Coef = −0.848, *p* = 0.018; Table 1). When the habitat score was low (lower tree density, higher wind speed, and noise level), other parid presence significantly increased the likelihood of the aggression response (Table S9 & Figure 1b).

**TABLE 1:** The best fit model results for response status using GLM (Generalized Linear Model; *n* = 183)

Variables	Coef.	*SE*	*z* value	Pr(>|*z*|)
(Intercept)	4.655	1.256	3.710	0.000
Aggression type	−3.159	0.796	−3.970	0.000
Other parids presence	−2.702	1.316	−2.050	0.040
Responding species	−0.335	0.721	−0.460	0.642
Larger hetero presence	−1.190	0.739	−1.610	0.107
Parid population	−0.032	0.130	−0.240	0.807
Vegetation/Wind/Noise	0.411	0.239	1.720	0.085
Hetero diversity	1.866	0.581	3.210	0.001
Aggression type * Hetero diversity	−0.923	0.560	−1.650	0.099
Other parids presence * Responding species	2.431	1.119	2.170	0.030
Other parids presence * Larger hetero presence	2.825	1.208	2.340	0.019
Other parids presence * Vegetation/Wind/Noise	−0.848	0.359	−2.360	0.018
Other parids presence * Hetero diversity	−0.825	0.447	−1.850	0.065

Note

Coef represents the estimated coefficients of each variable listed in the table. *SE* represents the standard error. * represents interaction terms.

### 3.2 Aggression level

**FIGURE 2:**
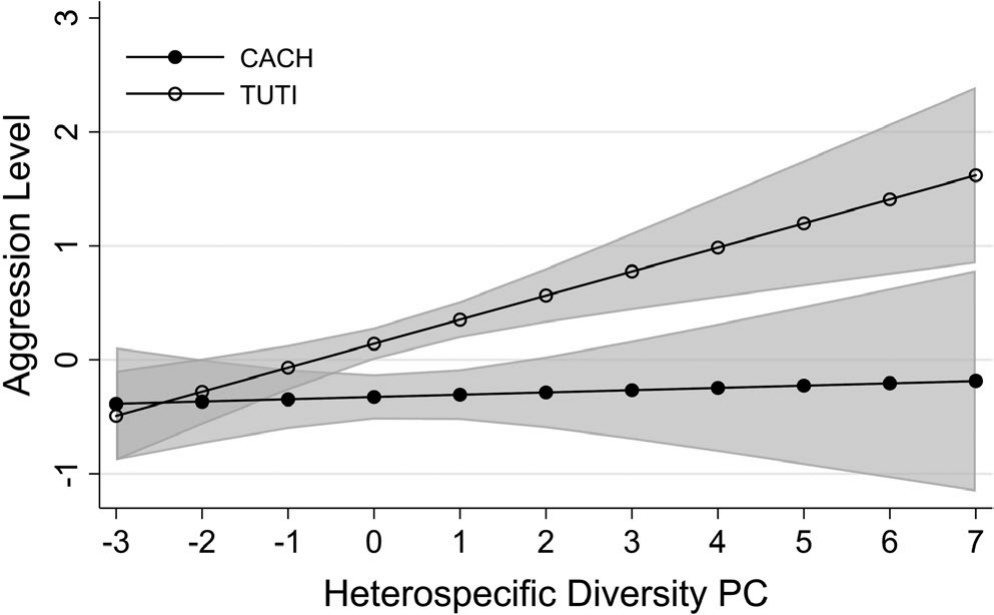
Marginal predicted values (and 95% CI) for aggression level defined by a two‐way interaction between responding species and the diversity of heterospecifics audiences observing the trial. Heterospecific diversity PC (*x*‐axis) represents principal component scores obtained by ordinating the number of species and individuals comprising the nonparid audience

Based on our best fit model of aggression level and the contrasts of predictive margins on significant interaction variables (Table 2 & Table S10), interspecific aggression was significantly lower than intraspecific aggression (Coef = −0.708, *p* <0.001; Table 2 & Figure S6) and TUTI showed significantly higher aggression levels than CACH (Coef = 0.469, *p* < 0.001; Table 2 & Figure S7). Aggression levels were significantly affected by an interaction between responding species and heterospecific diversity (Coef = 0.191, *p* = 0.030; Table 2). When the heterospecific diversity score was higher than −1 (range from −3 to 7), TUTI had a significantly higher aggression level than CACH (Table S10 & Figure 2).

**TABLE 2:** The best fit model results for aggression level using GLM (Generalized Linear Model; *n* = 139)

Variables	Coef.	*SE*	*z* value	Pr(>|*z*|)
(Intercept)	0.026	0.190	0.140	0.890
Aggression type	−0.708	0.113	−6.250	0.000
Other parids presence	−0.153	0.120	−1.280	0.201
Responding species	0.469	0.130	3.590	0.000
Larger hetero presence	0.026	0.150	0.170	0.862
Parid population	−0.036	0.035	−1.060	0.291
Vegetation/Wind/Noise	−0.042	0.043	−0.970	0.331
Hetero diversity	0.020	0.072	0.280	0.778
Responding species * Hetero diversity	0.191	0.088	2.160	0.030

Note

Coef represents the estimated coefficients of each variable listed in the table. *SE* represents the standard error. * represents interaction terms.

## 4 DISCUSSION

As expected, both interspecific and intraspecific aggression readily occurred in this study and the latter was significantly more probable and more intense, as expected from empirical and theoretical foundations (Tables 1and2; Kershner & Bollinger, 1999; Peiman & Robinson, 2010; Tynkkynen et al., 2004). We found strong support for our hypothesis that heterospecific audiences could influence respondents' aggression levels and both parids were more likely to respond to playback stimuli when their immediate audiences had higher heterospecific diversity (not including other parids). Indeed, when numerous other birds were in the audience, the probability of territorial contests became almost 100% (Figure 1a). We also detected asymmetric interaction effects of the presence of other parids on the probability of an aggressive response with specific conditions including larger heterospecific presence and variation in habitat conditions. Therefore, the number and kind of heterospecific species/individuals, the presence of other parids, and environmental conditions all influenced the probability of territorial aggression. These findings confirm emerging insights and expectations concerning impacts of heterospecific audiences on avian social interactions (Coppinger et al., 2020; Magrath et al., 2020). However, our work is novel in detecting strong heterospecific audience effects on aggression, a pervasive social interaction within free‐living bird communities not generally considered as being connected to heterospecific influences.

The role that aggression plays in structuring social groups and communities is relatively well known (Mac Nally & Timewell, 2005; Peiman & Robinson, 2010). However, the community richness context as it affects aggression among individuals is just beginning to be explored in animals (Kok et al., 2016; Makowicz et al., 2020), and studies of heterospecific audience effects on aggression are exceedingly rare (Thanh et al., 2005). This is due primarily to historical development of the topic, beginning with human sociology and then primarily sexual selection work animals; hence, the vast majority of studies on audience effects is limited to the conspecific context. Therefore, the distinct signature of heterospecific audience effects on aggression response probability and intensity we detected suggest further study may deepen understanding of animal community dynamics and potential conservation applications (Buchholz et al., 2019; Pillay et al., 2019; Tobias & Pigot, 2019).

### 4.1 Aggression intensity and response probability enhanced by a diverse audience

It is commonly known that animal species gather or travel together to share or compete for resources including services (e.g., vigilance and protection; Cords, 1990; Metcalfe, 1984; Sridhar et al., 2009) and goods (food/prey, information; Chen & Hsieh, 2002; DeStefano et al., 2000; Sridhar et al., 2009; Wellenreuther & Connell, 2002). But our findings invoke two key questions that are basically new. (1) Why should birds inspect or pause foraging to closely observe territorial conflicts (or playback in this case) of heterospecific neighbors and (2) why should combatants pay attention to variation in types of other species in the audience when engaging in contest or territorial behavior? To address (1) and ask what audience species might gain or accomplish by attending parid contests, we pose the following possibilities. One safe assumption is that the individuals gathered are familiar with one another, as the majority of species detected are nonmigratory and defend year‐round all‐purpose territories that overlap, including winter mixed‐species flocks (Jones et al., 2020; Jullien & Thiollay, 1998). Indeed, individual familiarity and frequent interaction set the stage for interspecific hierarchies to form, including positive associations among species (Hino, 2007), as well as potential tit‐for‐tat reinforcement of cooperative interactions (Krama et al., 2012). Given the proven values of situationally specific information provided by parids to sympatric species (Templeton & Greene, 2007), birds attending our trials may have been there to eavesdrop for useful information, or even to show support in some form for aid rendered by their local parids (Hetrick & Sieving, 2012; Huang et al., 2012; Jones & Sieving, 2019; Langham et al., 2006; Sieving et al., 2004, 2010). It is possible that some eavesdroppers might be attracted to aggression contest because they perceive the contest as a mobbing event or alarm calls. Heterospecifics may also gather to learn how valuable the fighting parids view their territories to be, based on their aggression level, as a means of assessing their own patch quality (Koops & Abrahams, 1999). The fact that local parids' judgment of patch quality is of interest to other species is a fundamental assumption underlying widespread heterospecific attraction to parids (Mönkkönen et al., 1999). Given territorial overlap, a vigorous conflict between sympatric parids may encourage collective boundary reinforcement by other species competing with adjacent conspecifics during breeding seasons (Munn & Terborgh, 1979). Therefore, it is possible that this type of multi‐species accretion at contests may represent one or more forms of local enhancement or behavioral synchronization (Gil et al., 2017).

Regarding (2) above, parid response probability increased when more heterospecific individuals and species were present during playback trials (Figure S8 shows how species and individual counts are related to the diversity PC scores on the *x*‐axis of Figure 1a). This pattern suggests that, as in mobbing aggregations, a higher heterospecific diversity of the mob increases the willingness of individuals to engage in conspicuous behavior (Krams et al., 2009). While some of the above factors may help explain the responding parid behaviors (e.g., information sharing, local enhancement), predation risk abatement is among the most compelling explanations for audience effects. Birds conduct every movement‐related decision under the constant threat of predation and engaging in aggressive conflict or territorial display enhances their detection by predators (Lima, 2009). For small forest birds like chickadees and titmice, both antipredator and territorial defense behaviors are conspicuous with frequent movements and calls, and these behaviors can attract raptors (Burnett & Sieving, 2016; Hua et al., 2016; Pereyra & Morton, 2010; Sieving et al., 2000). Aggression also distracts respondents from antipredator vigilance (Lima, 2009), which makes combatants highly susceptible to predation during the engagement. As a result, conspicuous combatants that perceive a possible attack will dampen their aggression intensity (Akçay et al., 2016). Therefore, it is reasonable to expect that a gathering of species around a fight provides many of the antipredator benefits that mixed‐species flocks and mobbing aggregations more generally provide to participants (shared vigilance, warning calls, dilution, etc.; Contreras & Sieving, 2011; Farley et al., 2008; Goodale et al., 2020; Jones & Sieving, 2019), thereby increasing the willingness of combatants to fight and the intensity with which they engage.

We have found consistent positive enhancement effects from diverse audiences on the probability of aggression response for both CACH and TUTI. However, diverse audiences only affect the aggression level of TUTI, not CACH (Figure 2). TUTI exhibited consistently higher overall aggression levels than CACH (Table 2). Thus, it is possible that CACH intruders are repelled at a lower level of aggression, and once the threshold is reached, even audience interest does not produce an audience‐driven increase in aggression. We also suggest the possibility that perhaps the presence of the large‐bodied birds does not reduce CACH risk perception as much as it does for TUTI, just because the CACH is so much smaller (~1/2 the mass of TUTI). While it is true that avian predators take the largest prey they can handle (Gotmark & Post, 1996; Malone et al., 2017), the smaller hawks and owls (*Accipiter* and *Otus* spp.) attracted to CACH calls would be less likely to successfully attack the much larger birds (Vézina, 1985). CACH are only 15% the size of a large woodpecker, whereas TUTI are over 20%. This difference may be enough to where the TUTI do, but the CACH do not, perceive greater safety from the presence of large species in the audience. We also found a significant positive interaction between other parid presence and larger heterospecific presence on aggression response, underscoring the positive feedback to TUTI aggression from more diverse audiences.

### 4.2 Complex relations between Carolina chickadees and tufted titmice

Modeling clearly showed that intraspecific aggression toward playback was more likely and more intense than interspecific aggression, presumably due to greater intraspecific niche overlap (Kershner & Bollinger, 1999; Peiman & Robinson, 2010). Also, TUTI exhibited higher aggression overall than CACH (both intraspecific and interspecific). Given that most interspecific aggressive interactions among closely related bird species are asymmetrical and favor the larger species (Martin et al., 2017), it is clear that the larger‐bodied TUTI is the dominant species (Cimprich & Grubb, 1994; Coppinger et al., 2020).

A notable finding, however, was that when TUTI were targeted in trials involving either treatment, titmice were more likely to respond and approach the playback if chickadees were also present during the playback trial. No such effect of TUTI presence on CACH was detected. This asymmetry is likely derived from the interspecies dominance relationship. In aviary trials involving antipredator versus control treatments in mixed flocks of TUTI and CACH, CACH vocal activity was repressed in the presence of TUTI to avoid aggression from the bigger birds in control conditions (without explicit predation risk or antipredator defense; Coppinger et al., 2020). In contrast, both TUTI and CACH called more in mixed flocks when presented with predation risk treatments, consistent with expectations under a social facilitation scenario during collective defense. We suggest that because TUTI are dominant to CACH, then in our trials, when TUTI are in a heightened state of aggression they might perceive attendant CACH as partners in social facilitation (of collective vigilance or defense of common territory) and increase their aggression in the presence of CACH without fear of conflict from them. Whereas when CACH are the aggressors, nearby TUTI are perhaps less likely to provide social facilitation for CACH than they are to attack them (Cimprich & Grubb, 1994; Contreras & Sieving, 2011).

### 4.3 Intertwined habitat quality and predation risk effects on aggression

Our analysis did, however, reveal one set of environmental conditions where the presence of either parid during intraspecific or interspecific playback treatments enhanced the likelihood of responding to playback, namely in open woodlands with low tree density and correlated higher levels of noise and wind speeds (Table S9; Figure 1b). We assume these conditions represent both lower habitat quality (less branch and leaf‐gleaning surface area for parid foraging; Jones et al., 2020) and higher raptor attack risk (less foliage cover, longer flights during foraging; Gentry et al., 2019; Grade & Sieving, 2016). If we assume that resources are poorer in these areas, then the presence of parids may trigger both heightened aggression to fight for limited resources (Johnson et al., 2004) and social facilitation to counteract predator attack during aggression. Indeed, overall aggressiveness may be higher where predation risk is higher (Dubois & Giraldeau, 2005). In contrast, most of our trial locations were in areas with larger, denser hardwood trees where food resources for both parids should be enriched (Jones et al., 2020) and predation risk perception may be lower because of the denser escape cover; both factors should decrease territorial aggression (Dubois & Giraldeau, 2005; Power & Conley, 1994).

### 4.4 A more inclusive definition of audience effects in animal ecology?

In conclusion, social interactions defined by the community context strongly influenced parid aggression. Specifically, we detected significant audience effects—heightened parid interspecific and intraspecific aggression—associated with higher diversity of heterospecific individuals present at simulated contests. Additionally, we detected an asymmetric effect of the presence of the related parid: TUTI exhibited a higher probability of aggressive response if CACH were present. We conclude that future studies quantifying aggression, particularly in free‐living animals, must consider the larger community context by assuming that heterospecific influences are likely. We show that the concept of audience effects, as they relate to avian aggression, should be expanded to incorporate the potential for diversity or state‐related heterospecific audiences. Thus, we propose that the expression of aggression in animal communities may have commonalities with more overtly interspecific collective behaviors such as flocking, predator‐mobbing, and eavesdropping networks driven by social information sharing (Gil et al., 2017).

## CONFLICT OF INTEREST

We have no conflicts of interest to disclose.

## AUTHOR CONTRIBUTION

**Jin Bai:** Conceptualization (equal); Data curation (equal); Formal analysis (lead); Investigation (lead); Methodology (equal); Project administration (lead); Resources (equal); Software (equal); Validation (equal); Visualization (supporting); Writing‐original draft (equal); Writing‐review & editing (equal). **Todd M. Freeberg:** Validation (equal); Writing‐review & editing (equal). **Jeffrey R. Lucas:** Validation (equal); Writing‐review & editing (equal). **Kathryn E. Sieving:** Conceptualization (equal); Data curation (equal); Funding acquisition (lead); Methodology (equal); Project administration (supporting); Resources (equal); Software (equal); Supervision (lead); Validation (equal); Visualization (lead); Writing‐original draft (equal); Writing‐review & editing (equal).

## Supporting information

Supplementary Material

## Data Availability

Data are available at https://ufdc.ufl.edu/IR00011304/00001.
