# Supplementary material for: A community context for aggression? Multi‐species audience effects on territorial aggression in two species of Paridae
Source: Ecol Evol. 2021 Apr 3;11(10):5305–19. doi: 10.1002/ece3.7421 (PMC8131767; doi:10.1002/ece3.7421)
Supplement: Supplementary file 1 — Supplementary Material [file ECE3-11-5305-s001.docx]

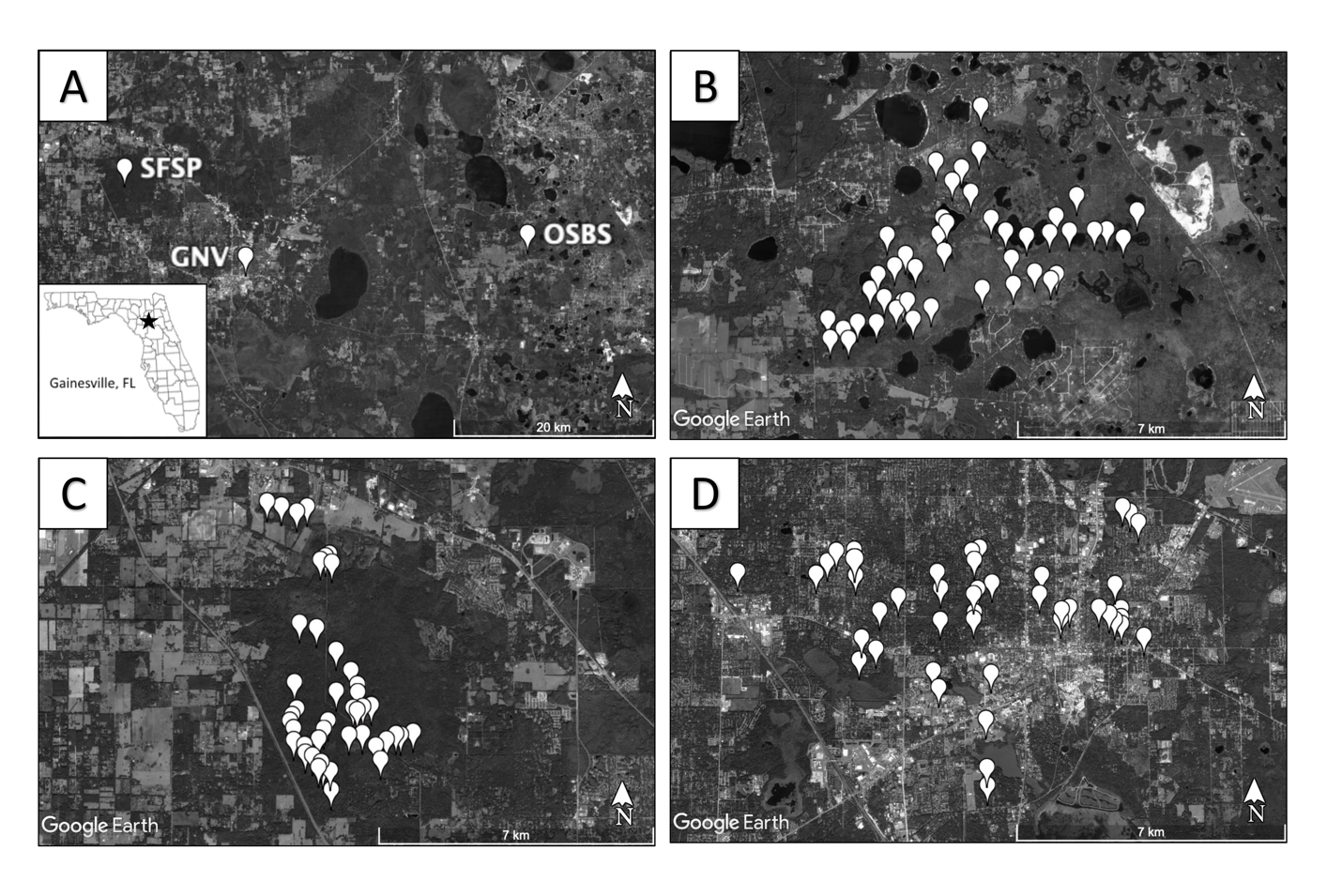


Figure S1. (A) Study site locations from Google Earth Pro. In each site, all playback trials are at least 200m away from each other; (B) Playback trials locations at Ordway-Swisher Biological Station (n=45); (C) Playback trials locations in San Felasco Hammock Preserve State Park (n=44); (D) Playback trials locations in the city of Gainesville (n=45).


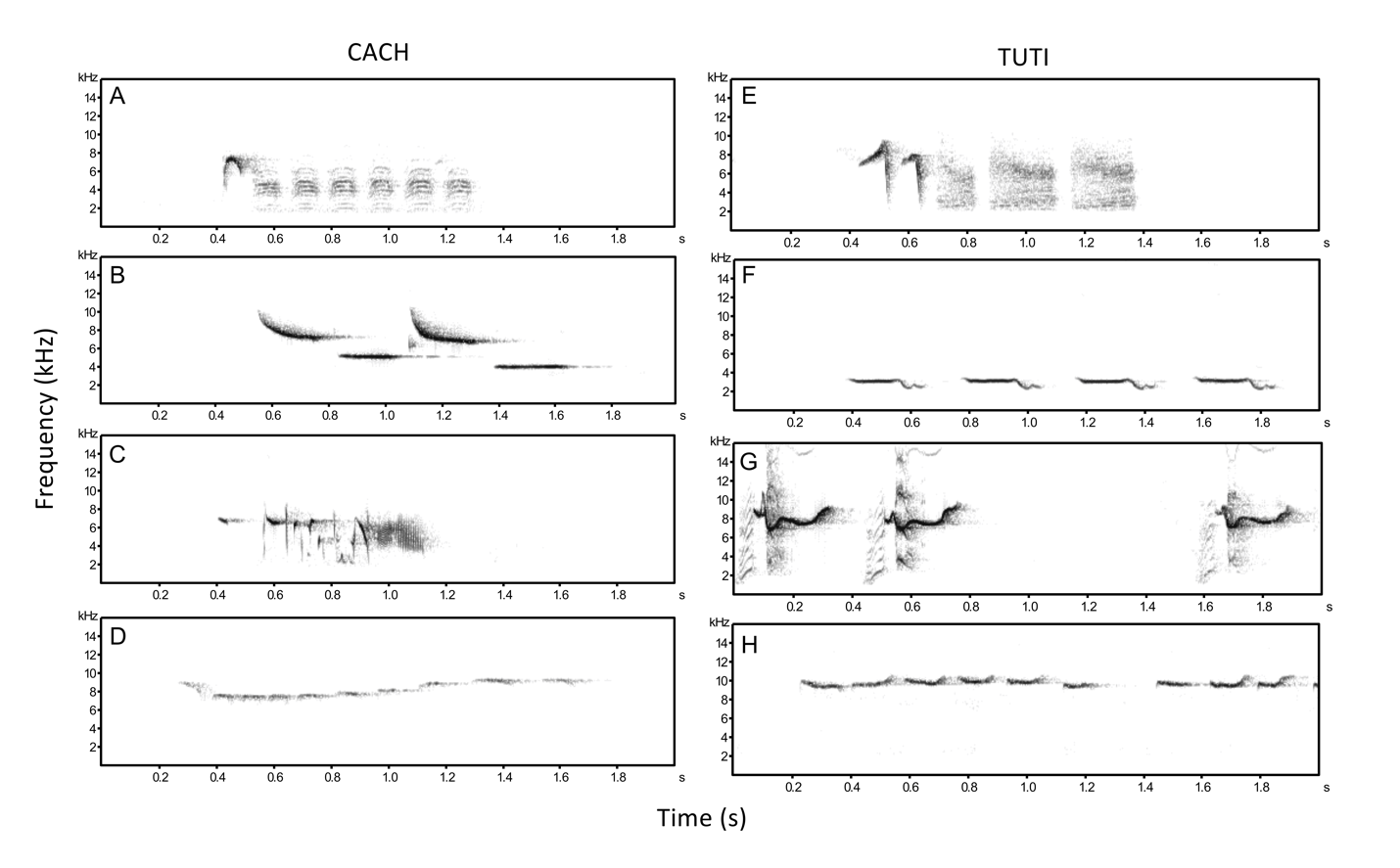


Figure S2. Spectrograms of Carolina chickadee vocalizations (A: chick-a-dee call; B: fee-bee song; C: gargle call; D: variable-see-strings call) and tufted titmouse vocalizations (E: chick-a-dee call; F: peter-peter song; G: squeal call; H: flutter display call).


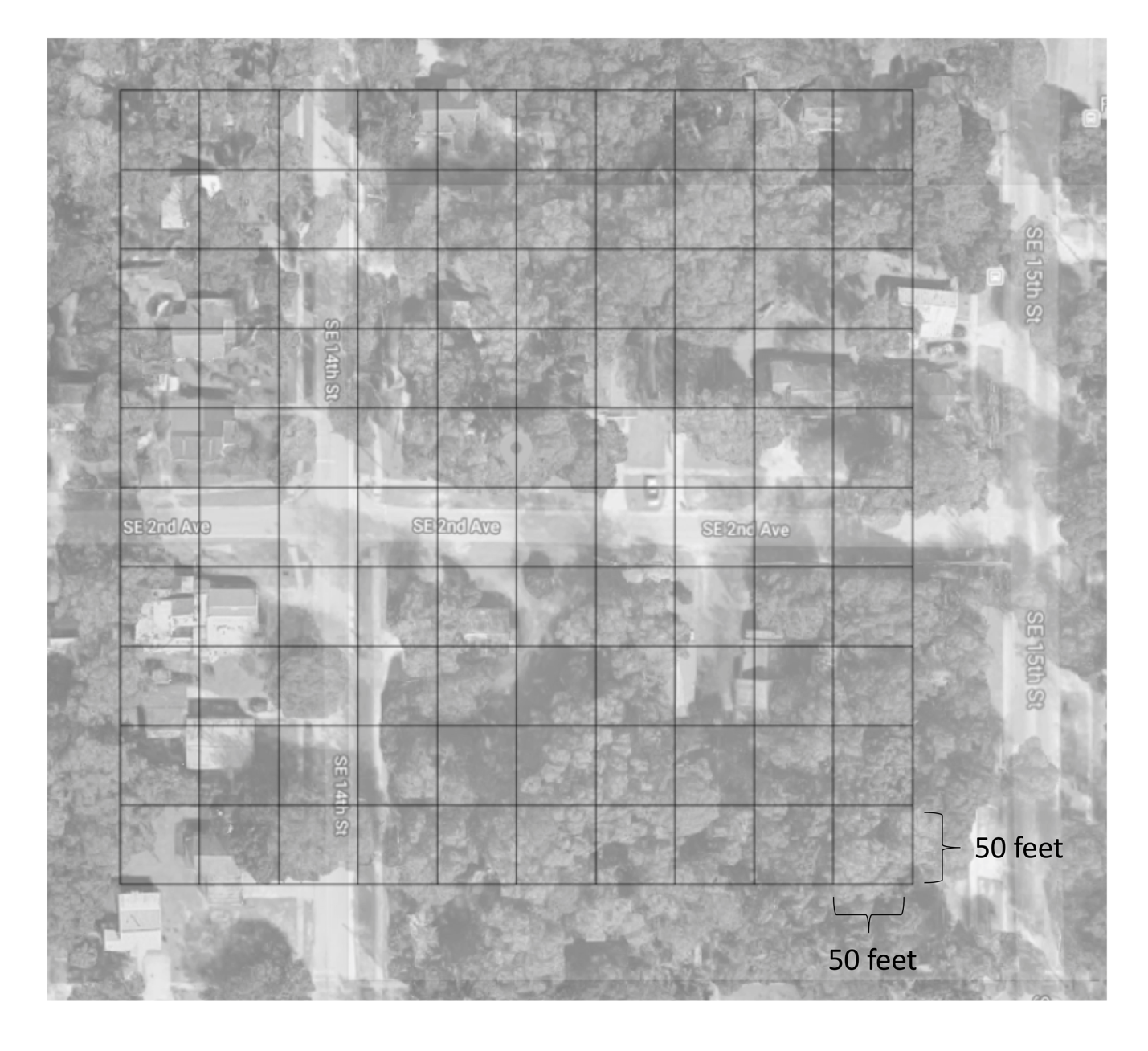


Figure S3. An example figure of canopy cover measurement. The playback trial location is at the center of the 10 by 10 quadrat. All Google images were captured at a scale of 350-meter aerial height. In each square, over 50% of the area covered by tree canopy signifies that the square has significant canopy cover. The percentage of canopy cover for each playback trial location was determined by the number of such squares in this 10 by 10 quadrat (number/100 total squares).


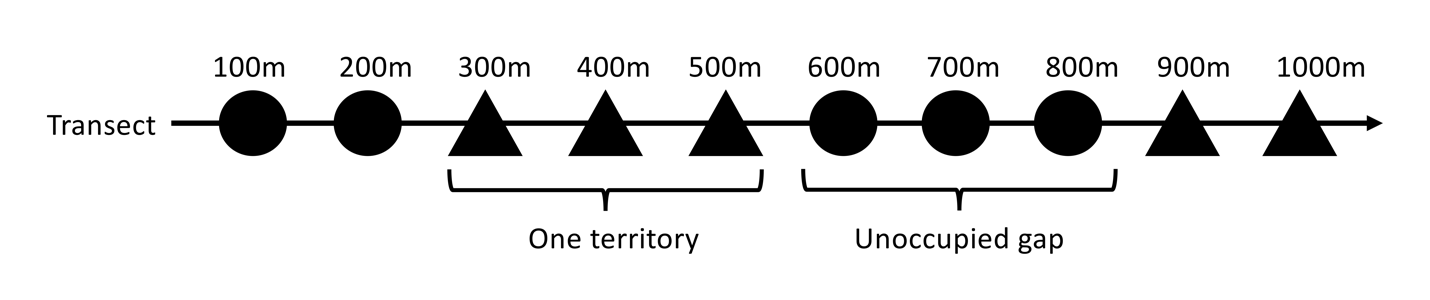


Figure S4. A schematic defining measures of territory density taken from transects sampled with playback at each 100m point along a 1 km transect. The circle represents a point without response, the triangle represents response, and every point is 100 meters apart. In this specific example, the territory density is 2 territories per transect.


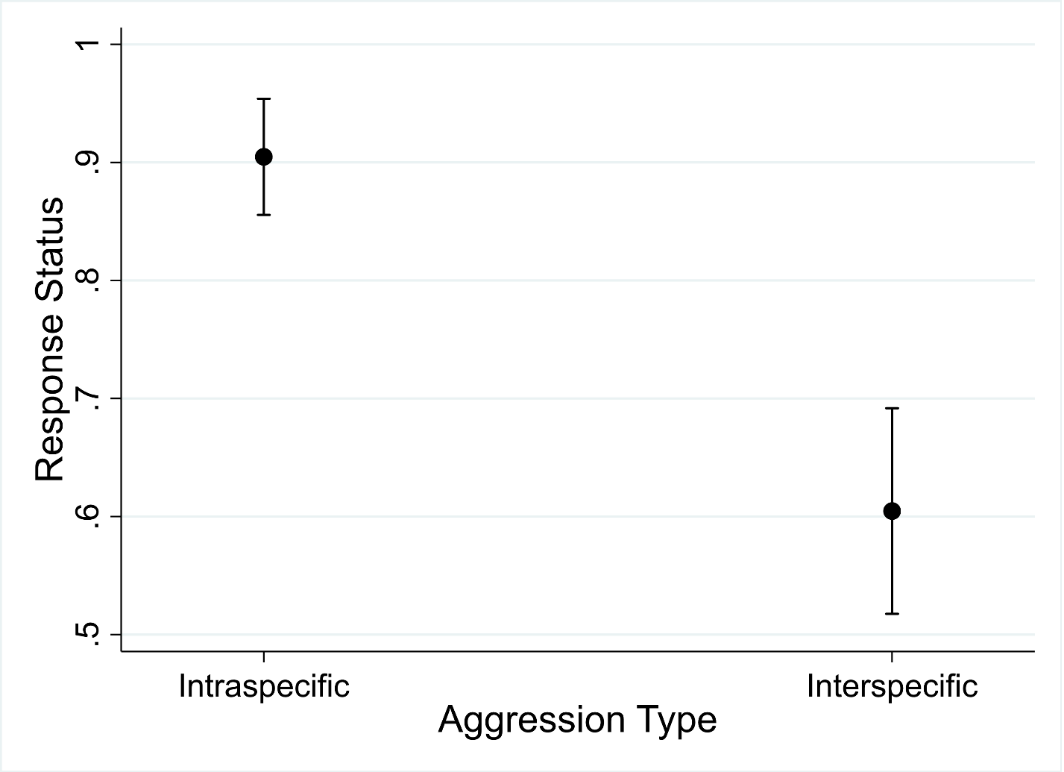


Figure S5. Predicted probability of response status at playback trials; the target species approached (1) or did not approach (0) as defined by aggression type (intra- versus inter-specific trial). Whiskers = 95% CI.


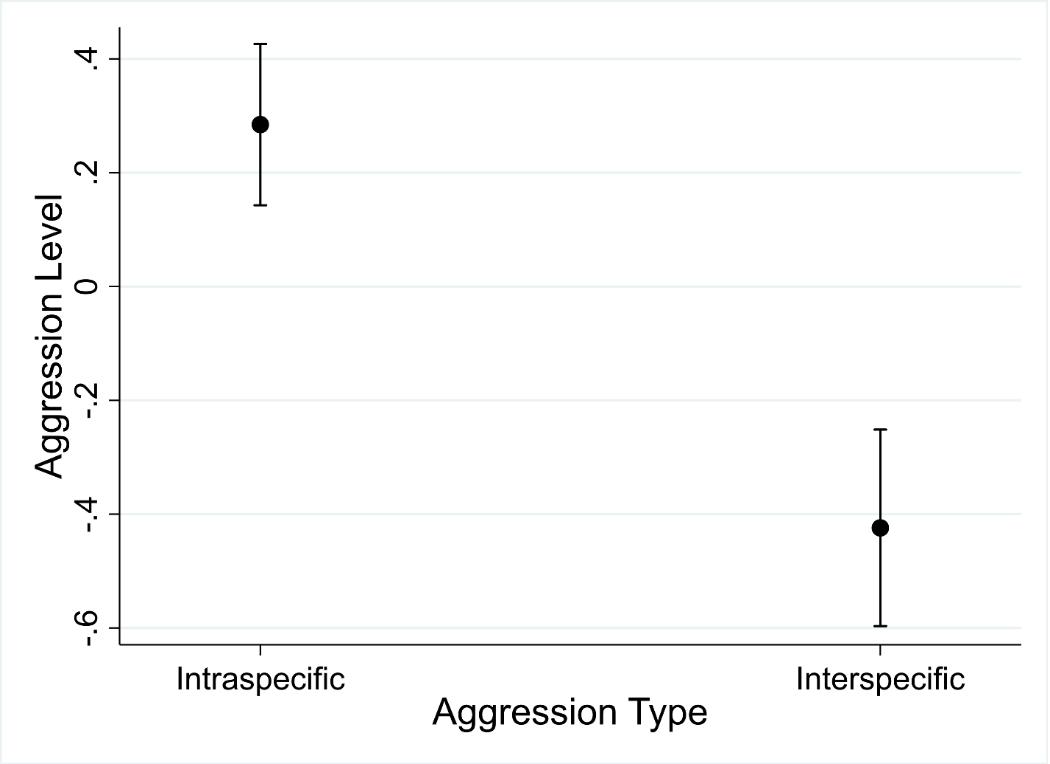


Figure S6. Predicted values for aggression level defined by aggression type (intra- versus inter-specific trial). Whiskers = 95% CI.


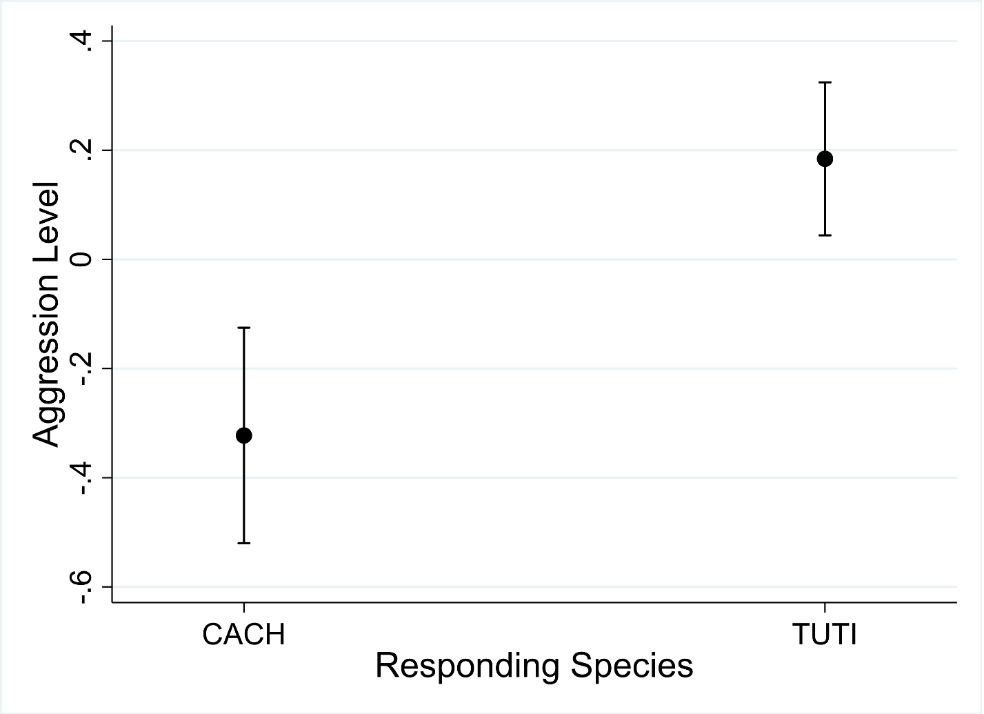


Figure S7. Predicted values for aggression level defined by responding species (CACH: Carolina chickadees; TUTI: tufted titmice). Whiskers = 95% CI.


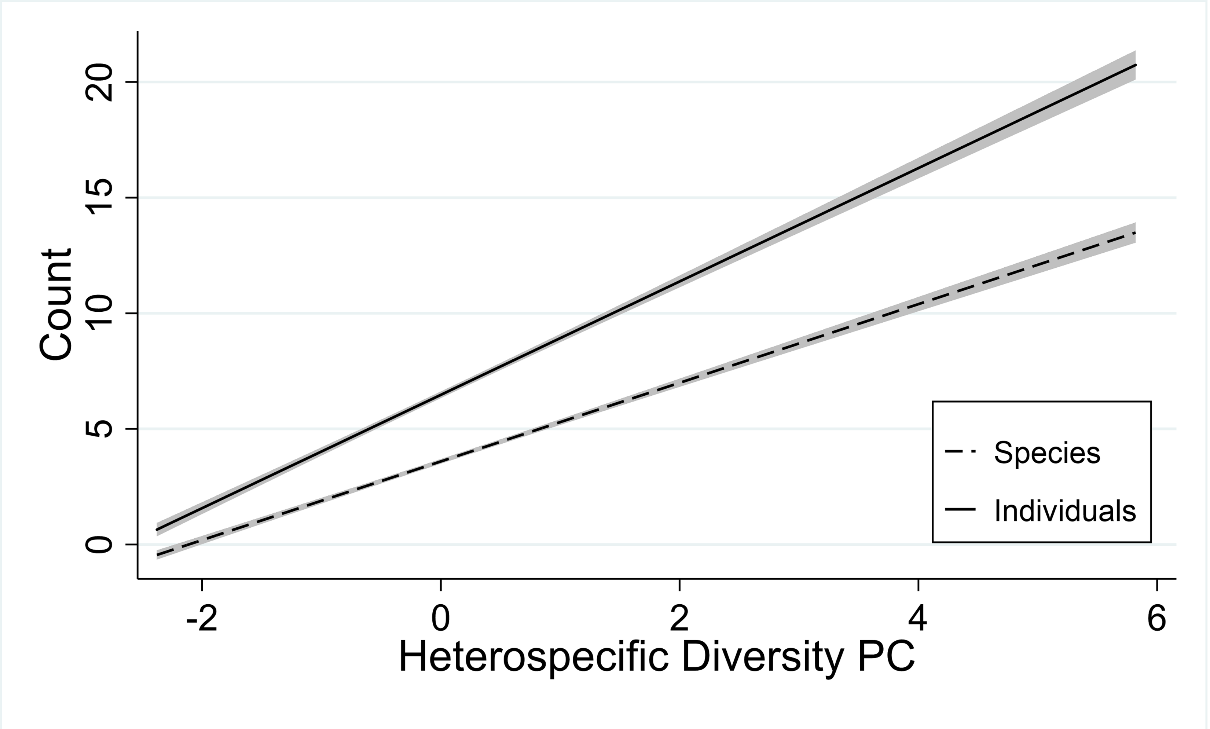


Figure S8. The relationship between the number of heterospecific individuals and the number of heterospecific species under the PCA score of heterospecific diversity.

Table S1. Organization of the aggression playback stimulus. Each intensity level consists of 2 mins playback for 8 mins total playback length. Placement of the calls into intensity classes are based on expert assessment (JAL, TMF, KES) as well as key references as noted in the text (Sieving et al., 2010; Pieplow, 2017).

| Intensity | CACH | TUTI |
| --- | --- | --- |
| Low | soft seets + short D sequence | soft seets + short D sequence |
| Mid | soft seets + fee-bee song | soft seets + peter-peter song |
| High | fee-bee song + gargles | peter-peter song + squeals |
| Max | long D sequence + fee-bee song  + gargles | long D sequence + peter-peter song + flutter display calls + squeals |

Table S2. The average number of calls were used in TUTI and CACH playback (each section is 2 mins tape). Sec. represents tape section sequence; D-note represents chick-a-dee call for both chickadees and titmice, peter represents titmouse song, squeal represents titmouse squeal call, flutter represents titmouse flutter display call; fee-bee represents chickadee song, gargle represents chickadee gargle call.

| TUTI | | | | | CACH | | |  |
| --- | --- | --- | --- | --- | --- | --- | --- | --- |
| Sec. | D-note | peter | squeal | flutter | D-note | fee-bee | gargle | |
| 1st | 19 | 0 | 0 | 0 | 25 | 0 | 0 | |
| 2nd | 0 | 27 | 0 | 0 | 0 | 21 | 0 | |
| 3rd | 0 | 25 | 38 | 0 | 0 | 23 | 22 | |
| 4th | 14 | 10 | 29 | 13 | 22 | 13 | 12 | |

Table S3. Top 10 most common species present (Paridae species are at the end of the table) during playback experiments. Counts represent the number of occurrences of each species across all playback experiments. (RBWO: Red-bellied Woodpecker; NOCA: Northern Cardinal; CAWR: Carolina Wren; DOWO: Downy Woodpecker; NOPA: Northern Parula; BLJA: Blue Jay; BGGN: Blue-gray Gnatcatcher; GCFL: Great-crested Flycatcher; NOMO: Northern Mockingbird; MODO: Mourning Dove; PIWO: Pileated Woodpecker; CACH: Carolina Chickadee; TUTI: Tufted Titmouse).

| Species code | Scientific name | Occurrences | Mean Mass (g) |
| --- | --- | --- | --- |
| RBWO | *Melanerpes carolinus* | 52 | 70.0 |
| NOCA | *Cardinalis cardinalis* | 49 | 45.0 |
| CAWR | *Thryothorus ludovicianus* | 37 | 19.0 |
| DOWO | *Picoides pubescens* | 26 | 30.0 |
| NOPA | *Setophaga americana* | 24 | 8.5 |
| BLJA | *Cyanocitta cristata* | 16 | 82.5 |
| BGGN | *Polioptila caerulea* | 9 | 6.0 |
| GCFL | *Myiarchus crinitus* | 7 | 32.5 |
| NOMO | *Mimus polyglottos* | 7 | 50.0 |
| MODO | *Zenaida macroura* | 5 | 130.0 |
| PIWO | *Dryocopus pileatus* | 5 | 275.0 |
| CACH | *Poecile carolinensis* | 67 | 11.0 |
| TUTI | *Baeolophus bicolor* | 116 | 20.0 |

Table S4. Summary of territory density in non-breeding seasons. A total of 3 km of transects were sampled once in SF and OS; a total of 6 km of transects were sampled once in UB; 1 km transects were separated by at least 500m. SF represents San Felasco Hammock Preserve State Park, OS represents Ordway-Swisher Biological Station, UB represents the city of Gainesville. All measurements in the table represent mean value among all 1 km transects within each study site. Values in the ( ) represent the range of the measurement.

| Species | Sites | Territory density (counts/km) | Gap distance (m) | Territory width (m) | Population density (counts/km) |
| --- | --- | --- | --- | --- | --- |
| TUTI | SF | 5 (3-6) | 150 (80-250) | 189 (167-200) | 12 (9-17) |
|  | OS | 3 (2-5) | 225 (100-400) | 164 (133-200) | 10 (7-12) |
|  | UB | 6 (5-8) | 127 (86-180) | 136 (120-150) | 16 (10-25) |
| CACH | SF | 4 (2-6) | 198 (120-250) | 131 (100-167) | 7 (4-10) |
|  | OS | 1 (0-1) | 900 (900-900) | 200 (200-200) | 1 (0-2) |
|  | UB | 6 (3-9) | 175 (80-350) | 142 (100-200) | 13 (6-19) |

Table S5. Summary statistics of each factor in aggression factor analysis.

| Factor | Eigenvalue | Difference | Proportion | Cumulative |
| --- | --- | --- | --- | --- |
| Factor1 | 2.280 | 1.528 | 0.808 | 0.808 |
| Factor2 | 0.753 | 0.534 | 0.267 | 1.075 |
| Factor3 | 0.218 | 0.252 | 0.077 | 1.152 |
| Factor4 | -0.034 | 0.141 | -0.012 | 1.140 |
| Factor5 | -0.175 | 0.045 | -0.062 | 1.078 |
| Factor6 | -0.220 | - | -0.078 | 1.000 |

Table S6. Factor loadings (pattern matrix) and unique variances. Cal_per_bird represents the number of calls per bird. Flt_per_bird represents the number of flights per bird. Lat_to_start (seconds) represents the latency to start responding to playback. Sec_in_20m (seconds) represents the time interval that the target bird spent responding to playback within 20 meters range. Closest_appr (meters) represents the closest approach distance of the target bird. Resp_grp_sz represents the response group size, which is the number of responding conspecific individuals.

| Variable | Factor1 | Factor2 | Factor3 | Uniqueness |
| --- | --- | --- | --- | --- |
| Cal_per_bird | 0.658 | 0.515 | 0.095 | 0.294 |
| Flt_per_bird | 0.697 | 0.180 | -0.230 | 0.429 |
| Lat_to_start | -0.486 | 0.161 | -0.174 | 0.708 |
| Sec_in_20m | 0.870 | -0.086 | 0.176 | 0.205 |
| Closest_appr | -0.500 | 0.194 | 0.306 | 0.619 |
| Resp_grp_sz | 0.346 | -0.620 | 0.038 | 0.495 |

Table S7. Summary statistics of aggression variables. Cal_per_bird represents the number of calls per bird. Flt_per_bird represents the number of flights per bird. Lat_to_start (seconds) represents the latency to start responding to playback. Sec_in_20m (seconds) represents the time interval that the target bird spent responding to playback within 20 meters range. Closest_appr (meters) represents the closest approach distance of the target bird. Resp_grp_sz represents the response group size, which is the number of responding conspecific individuals.

| Variable | Mean | Std. Dev. | Min | Max |
| --- | --- | --- | --- | --- |
| Cal_per_bird | 47.180 | 57.185 | 0.000 | 387.000 |
| Flt_per_bird | 8.540 | 8.661 | 0.333 | 65.000 |
| Lat_to_start | 168.777 | 149.940 | 7.000 | 939.000 |
| Sec_in_20m | 451.086 | 267.787 | 2.000 | 1064.000 |
| Closest_appr | 6.200 | 5.151 | 0.200 | 20.000 |
| Resp_grp_sz | 2.353 | 1.233 | 1.000 | 6.000 |

Table S8. Contrasts of predictive margins on the interaction variables (*p* < 0.05) for response status best fit model. Oth_prd_pres represents variable Other Parid Presence (present = 1, absent = 0). RespSpp represents variable responding Species (CACH = 1, TUTI =2). Lrge_het_pres represents variable Larger Heterospecific Presence (present = 1, absent =0).

| Variables | Contrast | Std. Err. | Chi2 | P>Chi2 |
| --- | --- | --- | --- | --- |
| RespSpp*Oth_prd_pres | - | - | - | - |
| (2 vs 1) * 0 | -0.044 | 0.091 | 0.230 | 0.632 |
| (2 vs 1) * 1 | 0.213 | 0.073 | 9.630 | 0.003 |
| Oth_prd_pres*RespSpp | - | - | - | - |
| (1 vs 0) * 1 | -0.066 | 0.100 | 0.430 | 0.511 |
| (1 vs 0) * 2 | 0.192 | 0.059 | 10.620 | 0.001 |
| Lrge_het_pres* Oth_prd_pres | - | - | - | - |
| (1 vs 0) * 0 | -0.139 | 0.073 | 3.610 | 0.057 |
| (1 vs 0) * 1 | 0.171 | 0.095 | 3.240 | 0.072 |
| Oth_prd_pres* Lrge_het_pres |  |  |  |  |
| (1 vs 0) * 0 | -0.114 | 0.102 | 1.240 | 0.265 |
| (1 vs 0) * 1 | 0.197 | 0.059 | 11.120 | 0.001 |

Table S9. Contrasts of predictive margins on the interaction variables (*p* < 0.05) for response status best fit model. Oth_prd_pres represents variable Other Parid Presence (present = 1, absent = 0). Habitat PC represents variable Vegetation/Wind/Noise Principal Component (positively correlated with basal area and canopy cover, negatively correlated with wind and noise levels; range from -3 to 3).

| Variables | Contrast | Std. Err. | Chi2 | P>Chi2 |
| --- | --- | --- | --- | --- |
| Oth_prd_pres * Habitat PC | - | - | - | - |
| (1 vs 0) * -3 | 0.381 | 0.115 | 10.920 | 0.001 |
| (1 vs 0) * -2 | 0.297 | 0.089 | 11.130 | 0.001 |
| (1 vs 0) * -1 | 0.206 | 0.066 | 9.770 | 0.002 |
| (1 vs 0) * 0 | 0.110 | 0.055 | 3.960 | 0.047 |
| (1 vs 0) * 1 | 0.011 | 0.066 | 0.030 | 0.866 |
| (1 vs 0) * 2 | -0.088 | 0.092 | 0.910 | 0.340 |
| (1 vs 0) * 3 | -0.184 | 0.122 | 2.250 | 0.134 |

Table S10. Contrasts of predictive margins on the interaction variables (*p* < 0.05) for aggression level best fit model. RespSpp represents variable responding Species (CACH = 1, TUTI =2). HetDiv represents variable Heterospecific Diversity (range from -3 to 7).

| Variables | Contrast | Std. Err. | Chi2 | P>Chi2 |
| --- | --- | --- | --- | --- |
| RespSpp * HetDiv | - | - | - | - |
| (2 vs 1) * -3 | -0.104 | 0.320 | 0.110 | 0.745 |
| (2 vs 1) * -2 | 0.087 | 0.241 | 0.130 | 0.719 |
| (2 vs 1) * -1 | 0.278 | 0.173 | 2.590 | 0.108 |
| (2 vs 1) * 0 | 0.469 | 0.130 | 12.920 | 0.000 |
| (2 vs 1) * 1 | 0.659 | 0.140 | 22.060 | 0.000 |
| (2 vs 1) * 2 | 0.850 | 0.195 | 19.040 | 0.000 |
| (2 vs 1) * 3 | 1.041 | 0.268 | 15.100 | 0.000 |
| (2 vs 1) * 4 | 1.232 | 0.348 | 12.530 | 0.000 |
| (2 vs 1) * 5 | 1.423 | 0.431 | 10.880 | 0.001 |
| (2 vs 1) * 6 | 1.614 | 0.516 | 9.770 | 0.002 |
| (2 vs 1) * 7 | 1.805 | 0.602 | 8.980 | 0.003 |
